# A fearful adult attachment style is associated with double the presence of chronic pain compared to secure attachment: A national survey of a South African population

**DOI:** 10.1111/bjhp.70024

**Published:** 2025-09-23

**Authors:** Gabriella Elisabeth Stamp, Stella Iacovides, Antonia Louise Wadley

**Affiliations:** ^1^ Brain Function Research Group, Department of Physiology, Faculty of Health Sciences, School of Biomedical Sciences University of the Witwatersrand Johannesburg South Africa

**Keywords:** attachment style, chronic pain, interpersonal relationships, pain burden, psychosocial

## Abstract

**Objectives:**

Preliminary epidemiological evidence suggests that within chronic pain cohorts, insecure attachment styles (comprising dismissing, preoccupied and fearful styles) are more prevalent. Our aim was to determine, in a general population, the association between adult attachment style and the presence and burden of chronic pain.

**Methods:**

A nationwide online survey in South Africa determined adult attachment style (using the Experience in Close Relationships—Relationship Structures Questionnaire), the presence of chronic pain and typically associated psychological factors. In participants reporting chronic pain, the association with attachment style and pain burden (pain sites, severity and interference, using the Brief Pain Inventory [BPI]) was further explored. Results of the 2371 participants were analysed using multivariable generalized linear models.

**Results:**

In our young (median age 23 years; IQR 20–28), well‐educated and primarily female (74%) cohort with a predominantly middle‐to‐high socioeconomic status, we found a higher than typically reported prevalence of chronic pain (27%). Compared to the secure attachment style, all insecure attachment styles were associated with increased chronic pain presence (secure: 23%; dismissing: 31%, odds ratio [95% CI] = 1.38 [1.02–1.85], *p* = .037; preoccupied: 42%, odds ratio [95% CI] = 2.26 [1.62–3.13], *p* < .001; fearful: 49%, odds ratio [95% CI] = 2.95 [2.03–4.29], *p* < .001). All three insecure attachment styles were associated with worse pain interference, and a Fearful attachment style was associated with increased pain severity and .78 times more pain sites (95% CIs: not spanning 0, *p*s < .05).

**Conclusions:**

Adult attachment style was associated with chronic pain presence and pain burden. The presence of chronic pain was more than double in the fearfully insecure compared to securely attached individuals.

**Statement of contribution**

**What is already known on the subject?**

- In chronic pain cohorts, insecure attachment styles are more prevalent than a secure style.
- In population‐based studies, insecure attachment styles have been associated with the presence of subsects of chronic pain.
- Studies investigating associations between attachment style and pain burden have produced equivocal results.

**What does this study add?**

- Insecurely attached South Africans were more likely to have chronic pain than those who were securely attached.
- Fearfully attached individuals had more than double the chronic pain presence of securely attached individuals.
- Fearfully attached individuals had increased pain severity, interference, and number of pain sites.

## INTRODUCTION

Chronic pain is defined as pain lasting for more than 3 months (Treede et al., 2015) and is the leading cause of disability globally (James et al., 2018). Biological, psychological, and social factors associate with chronic pain and its experience (Nicholas, 2022): Biological factors, including increased inflammation (Iordanova Schistad et al., 2020); psychological factors, such as depression and anxiety (Aaron et al., 2025; De La Rosa et al., 2024), exposure to trauma (Nicol et al., 2016), and appraising pain as threatening (pain catastrophizing) (Severeijns et al., 2001); and social factors, including lower socioeconomic status and social support (Khalatbari‐Soltani & Blyth, 2022; Oraison & Kennedy, 2021).

As the global prevalence of chronic pain is predicted to increase (Zhu et al., 2025), predicting who develops chronic pain is important for planning resource allocation and targeted prevention strategies (Blyth et al., 2019; Delgado‐Sanchez et al., 2023; Foley et al., 2021). Multiple theoretical models exist to explain vulnerability to developing chronic pain including the fear avoidance model (Lethem et al., 1983; Vlaeyen et al., 2016), the biopsychosocial approach of chronic pain (Engel, 1977; Gatchel et al., 2007; Miaskowski et al., 2020), and the attachment‐diathesis model of chronic pain (Meredith et al., 2008). These models, and others, are critically and comprehensively reviewed by Delgado‐Sanchez et al. (2023), who conclude that these psychological theories are valuable, particularly for identifying the psychological characteristics that make people vulnerable to developing chronic pain. This review (Delgado‐Sanchez et al., 2023) and another on the role of psychosocial factors in the development and maintenance of chronic pain (Edwards et al., 2016), point out, however, that these models do not link psychological characteristics to biological correlates well. In a recent interdisciplinary review, we used neurobiological and psychobiological literature to describe the biological correlates and mechanisms that may link social connections, and in particular attachment styles, to chronic pain development (Stamp et al., 2024).

**FIGURE 1:**
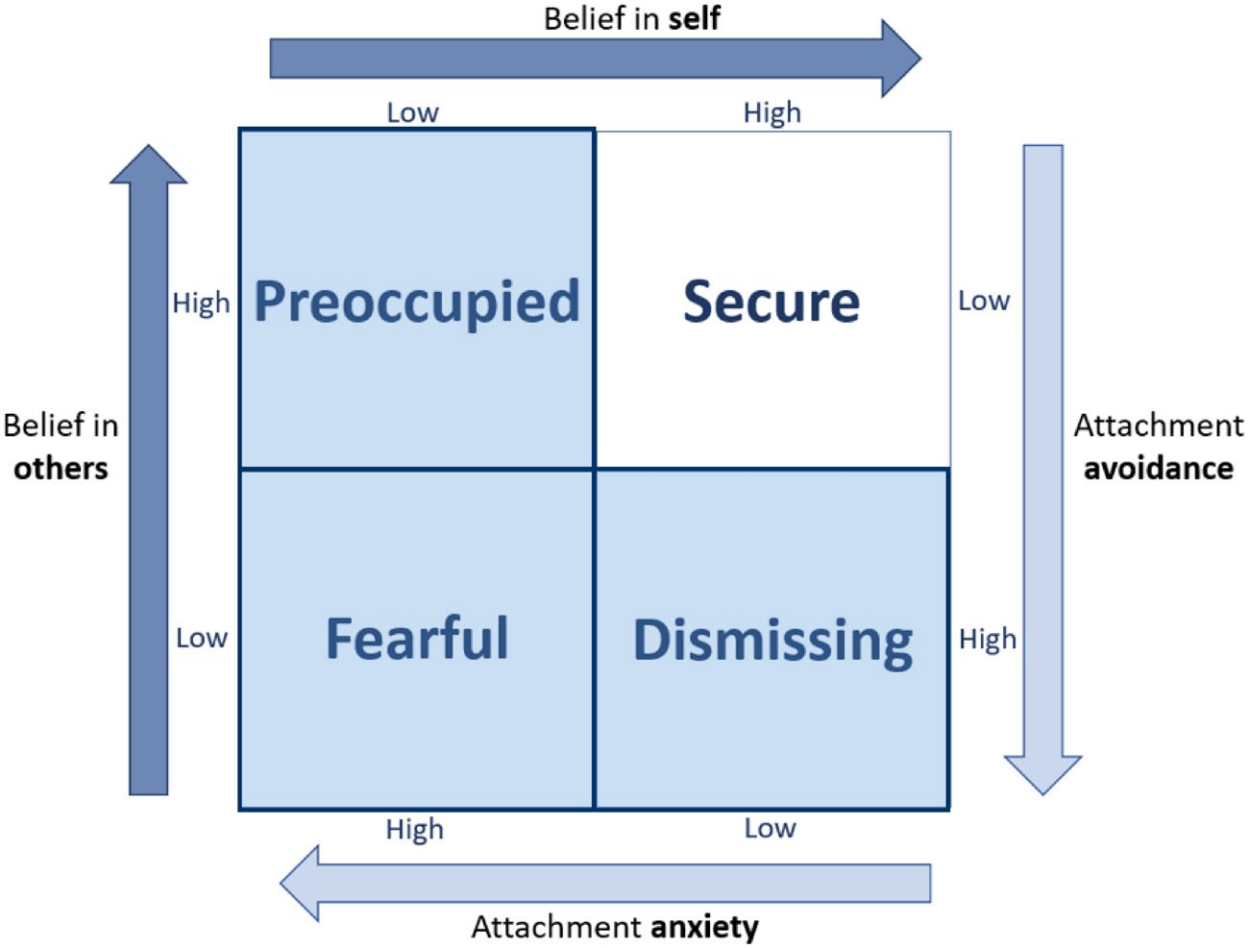
The four adult attachment styles based on the model of adult attachment described in (Bartholomew & Horowitz, 1991). The three highlighted quadrants are all insecure attachment styles.

The attachment‐diathesis model suggests that since attachment style serves as a framework for one's appraisals and perceptions, when one is exposed to a painful stimulus, the attachment style or internal working model is activated (diathesis) (Meredith et al., 2008). Since attachment style describes an individual's trust in themselves and others during times of stress (Bartholomew & Horowitz, 1991; Mikulincer & Shaver, 2007), the nature of the attachment style (secure or insecure, comprising dismissing, preoccupied and fearful; Figure 1) will consequently shape appraisals about pain, one's own ability to manage it, and the perceived ability of others to provide support. The appraisals will, in turn, determine the individual's emotional state, coping strategies and support‐seeking behaviours, which will ultimately affect how one experiences and manages pain, and also who may go on to develop chronic pain following acute pain.

Theoretically, individuals with an insecure attachment may be more likely to develop chronic pain, as there are overlapping comorbidities and risk factors including depression and anxiety (Ciechanowski et al., 2003), exposure to traumatic life events (Nacak et al., 2017), greater inflammation (Jaremka et al., 2013) and disrupted sleep (Adams & McWilliams, 2015). Individuals may also be less likely to effectively manage chronic pain, due to less adaptive appraisals and coping strategies such as higher pain catastrophizing, lower self‐efficacy, fear avoidance and hypervigilance, often adopted or experienced by individuals with insecure attachment (Andersen, 2012; Ciechanowski et al., 2003; Kowal et al., 2015; McWilliams & Holmberg, 2010; Meredith et al., 2007). This theory is supported by studies in various chronic pain populations in children, adolescents and adults (Carotenuto et al., 2013; Laird et al., 2015; Meredith et al., 2005; Nacak et al., 2017; Peñacoba et al., 2018; Savi et al., 2005). Indeed, population‐based studies of attachment and pain have found associations between having an insecure attachment style and chronic widespread pain (Davies et al., 2009) and medically unexplained pain (McWilliams, 2017), which are two subsects of chronic pain; and chronic pain when defined using a non‐standard question with an unclear timeframe (McWilliams & Bailey, 2010). In studies *within* chronic pain populations, insecure attachment styles are either more common, for example, in a group of 235 individuals presenting for a pain management programme, where 66% had an insecure attachment style (Kowal et al., 2015); or more prevalent than in healthy controls. For example, in 101 headache clinic patients attending a neurology clinic, two‐thirds had an insecure adult attachment style (66%, 34/99) compared to half in healthy controls (50%, 2227/4454) (Belot et al., 2021). No study has explored in a healthy population the associations between attachment style and chronic pain, using a standard definition.

Studies investigating the association between attachment style and pain intensity and disability have produced equivocal results (Andersen, 2012; Davies et al., 2009; Kowal et al., 2015). No association has been found between pain intensity and fearful, dismissing and preoccupied styles (Ciechanowski et al., 2003; Davies et al., 2009). Disability (also understood as pain‐related interference) has not been associated with fearful or dismissing styles, and there have been equivocal results for the preoccupied style (Ciechanowski et al., 2003; Davies et al., 2009). Only one study has investigated the association between the number of pain sites in chronic widespread pain and attachment style, and found a greater number of pain sites in individuals with fearful, dismissing and preoccupied styles (Davies et al., 2009).

Although the attachment‐diathesis model seems robust and is supported by various data in populations with chronic pain, the effect sizes are sometimes small (Andersen et al., 2019; Delgado‐Sanchez et al., 2023). Thus, in this study, we conducted a large online survey of a general South African population to determine if the presence of chronic pain was associated with adult attachment style. Secondly, in those with chronic pain, we investigated whether pain burden—comprising pain intensity, interference and number of pain sites—was associated with attachment style. As the closest related study to ours used subtypes of insecure attachment (Davies et al., 2009), we decided to do the same so that we could capture the nuance of those with both high anxiety and high avoidance dimensions. We hypothesized that individuals with insecure attachment styles of all subtypes (preoccupied, dismissing and fearful) would be more likely to have chronic pain, and that that pain would be more intense, at a greater number of pain sites and cause greater pain interference.

## MATERIALS AND METHODS

### Ethical clearance

The study protocol was approved by the Human Research Ethics Committee (Medical) at our South African university, which adheres to the principles of the Declaration of Helsinki and the Declaration of the World Medical Association (clearance certificate number: M210449).

### Participants

Male and female individuals aged 18 years and older, who were born in, and currently residing in, South Africa, were invited to participate in an online survey. The survey introduction and link were distributed via social media platforms and university emails at the University of the Witwatersrand, University of the Free State, and the University of Cape Town, all of which are in South Africa. The survey was conducted between September 2021 and April 2022.

### Survey

The survey consisted of customized questionnaires that assessed demographic and socioeconomic factors, including age, race, sex, education and annual household income. Socioeconomic status was inferred by the reported annual household income. Participants were categorized into low (ZAR 1–ZAR 19200 ~ USD 0.6–USD 1095), middle (ZAR 19201–ZAR 307200 ~ USD 1095–USD 17513) and high income (>ZAR 307200 ~ >USD 17513) categories (Statistics South Africa, 2011). We also assessed psychosocial factors and pain‐related variables using other standardized questionnaires. As detailed below, the psychological factors assessed included adult attachment style, stress, anxiety and depression (using the Experience in Close Relationships—Relationship Structures Questionnaire, and the Depression, Anxiety and Stress Scale 21, respectively). The pain‐related variables that were assessed included pain catastrophizing (using the Pain Catastrophizing Scale Questionnaire), and to assess pain burden in participants reporting chronic pain, the Brief Pain Inventory—Short form questionnaire was used to determine the severity of pain, location of pain, number of pain sites, and pain interference.

The survey was created and disseminated using Redcap, a secure, web‐based software platform designed to support data capture for research studies (Harris et al., 2009, 2019) and hosted at the University of the Witwatersrand. The platform does not suggest responses, and so potential automated responses were controlled for. As individuals could leave their email address at the end if they were interested in taking part in a future study, responses were confidential but not necessarily anonymous. There was no compensation for taking part.

### Adult attachment style

Adults have a network of attachment figures that can vary throughout adulthood (Doherty & Feeney, 2004; Tian & Freeman, 2024). As we planned to recruit a mostly student sample who may not yet be in long‐term partnerships, we used the Experience in Close Relationships – Relationship Structures (ECR‐RS) questionnaire to assess adult attachment style (Fraley et al., 2011), which measures attachment with the four most common attachment figures in adulthood (rather than focusing on a single attachment figure). The ECR‐RS was adapted from the Experiences in Close Relationships—Revised questionnaire (Treede et al., 2015) and shares a smaller number of the same questions. The ECR‐RS is a 9‐item questionnaire with a 6‐item subscale for avoidance and a 3‐item subscale for anxiety, and has been previously validated in a non‐clinical sample (Fraley et al., 2011), although not in South Africa. The 9 items are repeated four times, each with respect to a different relationship (a relationship with a mother/mother‐like figure, father/father‐like figure, romantic partner, and best friend). The questionnaire is scored on a 7‐point Likert scale anchored at 1 (representing “strongly disagree”) to 7 (representing “strongly agree”). The mean scores for the six avoidance items and three anxiety items for each relationship were calculated, and the mean of these scores over the four relationships gave the global dimensions for attachment anxiety and avoidance (Fraley et al., 2011). The global dimensions were then classified into adult attachment styles based on the 4‐category model of attachment (Bartholomew & Horowitz, 1991) by splitting both the dimensions in half. Thus, a score of ≤4 for both attachment anxiety and avoidance dimensions was classified as a secure adult attachment style, while a score of >4 for both attachment dimensions was classified as a fearful adult attachment style. A score ≤4 for the attachment anxiety dimension but >4 for the attachment avoidance dimension was classified as a dismissing adult attachment style. Lastly, a score ≤4 for the attachment avoidance dimension and >4 for the attachment anxiety dimension was classified as a preoccupied adult attachment style. Figure 1 shows the interactions between the dimensions and how attachment anxiety equates to belief in self and attachment avoidance to belief in others (Bartholomew & Horowitz, 1991; Mikulincer & Shaver, 2007).

### Depression, anxiety and stress

The Depression, Anxiety and Stress Scale 21 (DASS‐21) was used to measure depression, anxiety, and stress (Lovibond & Lovibond, 1995). The DASS‐21 has been validated in a South African non‐clinical population (Dreyer et al., 2019) and has been found to be an effective measure of depression in patients with chronic pain due to the absence of questions about somatic symptoms (Taylor et al., 2005). The DASS‐21 is a 21‐item questionnaire with three 7‐item subscales assessing depression, anxiety, and stress. The questionnaire was scored on a 4‐point Likert scale anchored at 0 (representing “never” applies to the individual) to 3 (representing “almost always” applies to the individual). The total score for each subscale was 21, with scores above 13, 9 and 16 indicating extremely severe depression, anxiety and stress, respectively.

### Pain catastrophizing

The Pain Catastrophizing Scale (PCS) questionnaire was used to measure the participant's tendency to adopt pain catastrophizing thoughts. The PCS is a 13‐item questionnaire assessing pain rumination, pain magnification and helplessness, and has previously been validated in a non‐clinical sample (Sullivan et al., 1995) and in a South African sample with fibromyalgia (Morris et al., 2012). The PCS was scored on a 5‐point Likert scale anchored at 0 (representing “not at all”) to 4 (representing “all the time”). The highest possible score for the PCS questionnaire is 52 (Sullivan et al., 1995).

### Chronic pain

Participants were asked, “Have you had pain most days for the past three months?” Those responding ‘yes’ were classified as having chronic pain. This simple definition of chronic pain (pain experienced most days for the last 3 months) is used in the 11th, and most recent, revision of the WHO's International Classification of Diseases (Treede et al., 2015). It covers pain of all causes, including primary pain, that is, pain with no identifiable cause (fibromyalgia and non‐specific low back pain), and secondary pain, where pain is a symptom of another condition or illness (Treede et al., 2015). Participants who reported having chronic pain answered the Brief Pain Inventory—Short form (BPI‐sf) questionnaire (Cleeland & Ryan, 1994), which was used to determine pain severity, location of pain, number of pain sites, and pain interference. The questions were scored on a numerical rating scale (NRS) anchored at 0 (no pain) to 10 (worst pain imaginable). A total pain severity score was calculated by finding the mean of the reported pain intensities of the participants' worst, least, average and current pain over the last week (Cleeland, 2009). Similarly, the pain interference score was calculated by finding the mean of reported interference of pain in general activity, mood, walking, work, relations with people, sleep, and enjoyment of life (Cleeland, 2009). Whilst the BPI has not been validated in this population, the Wisconsin brief pain questionnaire (Daut et al., 1983), which is essentially the same as the BPI except for one timeframe (asking about pain in the last month and not the last week), has been validated in a South African population (Mphahlele et al., 2008).

### Data analysis

Before determining whether chronic pain was associated with attachment style, we first wanted to ensure we had sufficient coverage of all attachment styles in the survey. Whilst the least frequent attachment style differs between studies and populations, the least frequent attachment style has had a prevalence ranging from 4% to 13% (Bakermans‐Kranenburg & van IJzendoorn, 2009; Coe et al., 1995; Davies et al., 2009; Díaz‐Mosquera et al., 2022). A sample size analysis (Bartlett et al., 2001) determined that a minimum sample size of 2305 was required to ensure that we had a representative sample that could detect a 4% prevalence of a style and could answer the question of whether adult attachment style is associated with the presence of chronic pain in a general South African population with a 95% confidence level of certainty.

Descriptive data are reported as total number (N) and percentages (%) for categorical data and median [interquartile range (IQR)] for skewed continuous data. We used variations of generalized linear models for our inferential analyses. For our primary analysis (the relationship between adult attachment style and chronic pain presence), a univariate logistic regression model was run for adult attachment style with chronic pain, using incidence rate ratios to describe the relationship. Thereafter, a multivariable logistic regression model was run to control for covariate and confounding factors for chronic pain, excluding collider variables. For our secondary analysis looking at the relationship between adult attachment style and the measures of the burden of chronic pain (chronic pain severity, interference and number of pain sites), univariate linear regression models (adult attachment with pain severity, interference, or pain sites) were run. Subsequently, multivariable linear regression models (including covariates and confounders) were run for each measure of the burden of pain. Lastly, a dropout analysis was performed to compare the attachment styles, gender, annual household income, age, depression, anxiety and pain catastrophizing of individuals who reported chronic pain but did not complete the BPI and those who reported chronic pain and completed the BPI. The dropout analysis was for the purpose of identifying possible reasons for lack of completion of the BPI questionnaire.

**FIGURE 2:**
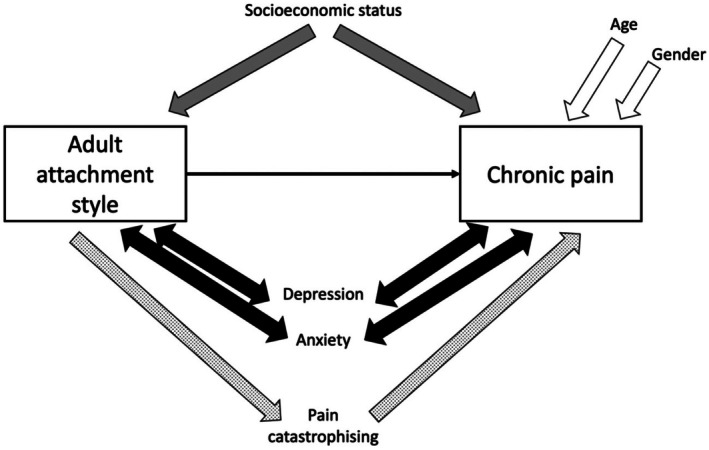
Types of variables analysed in the study, and how each of them was accounted for in the multivariable analyses. To determine the total effect of the independent variable (adult attachment style) on the outcome variable (chronic pain), both confounders and covariates should have been accounted for in a multivariable analysis. Age and gender have been previously found to affect chronic pain (white arrows), making them covariate factors (Kamerman et al., 2020) and so were included. Socioeconomic status has been found to affect adult attachment style (Sakman et al., 2022) and chronic pain (Prego‐Domínguez et al., 2021) (grey arrows), making it a confounding factor, and so was also included. Since there are bi‐directional relationships with depression and anxiety, and both the independent variable (attachment) and the dependent variable (chronic pain) (Humo et al., 2019; Rajkumar, 2022; Yamauchi et al., 2022) (Golshani, 2021) (black arrows), depression and anxiety are both classified as collider variables and were removed from the multivariable model (MacKinnon & Lamp, 2021). Lastly, adult attachment style has been found to affect pain catastrophizing (McWilliams & Holmberg, 2010), which in turn, has been found to affect chronic pain (Severeijns et al., 2001) (pattern arrow), making it a possible mediating factor. Mediators should also be excluded from a model when looking for the total effect of the independent variable on the outcome variable (MacKinnon & Lamp, 2021), and so we excluded pain catastrophizing in the multivariable model.

Figure 2 illustrates the different types of variables to explain why variables were included or excluded in the multivariable models. Data are reported as odds ratios and crude estimates (95% confidence intervals [CI]) for the logistic regressions, incidence rate ratios and crude estimates (95% [CI]) for Poisson regressions and estimates with 95% [CI] for linear regressions. All data processing and analysis were performed in the R statistical environment (v4.2.1) (R Core Team, 2022), using the following packages: DHARMa (Hartig, 2022), emmeans (Lenth, 2022), lme4 (Bates et al., 2015), pscl (Jackman, 2020), psych (Revelle, 2022), sjPlot (Lüdecke, 2022), tidyverse (Wickham et al., 2019) and VGAM (Yee et al., 2015). A *p*‐value <.05 was considered to be statistically significant for all analyses. All data, analysis scripts and analysis script outputs are available at Figshare: https://doi.org/10.6084/m9.figshare.23531076.v1.

## RESULTS

Overall, there were 3356 survey entries, of which 2371 individuals completed the entire survey and were included in the statistical analyses. The age range of the participants was 18–80 years old, with a median [IQR] of 23 [20–28] years. At least secondary education had been completed by 99% (2362 of 2371) of the participants, and 79% (1877 of 2371) of the participants had an annual household income of greater than ZAR19 200,00 (considered as middle and high income households (Statistics South Africa, 2011)). Almost two‐thirds of respondents were students, and a third were employed (either full‐time, part‐time or self‐employed). The majority of respondents were single and had never been married. The prevalence of chronic pain was 27% (635 of 2371) in our sample. More details of our survey cohort are described in Table 1.

**TABLE 1:** A summary of the demographic data and adult attachment style proportions in the 2371 survey respondents.

Characteristic	Final sample, *N* (%)
Gender	
Men	596 (25)
Women	1747 (74)
Other	28 (1)
South African Province	
Eastern Cape	53 (2)
Free State	382 (16)
Gauteng	1277 (54)
Kwa‐Zulu Natal	113 (5)
Limpopo	43 (2)
Mpumalanga	37 (1)
Northern Cape	14 (1)
North West	28 (1)
Western Cape	424 (18)
Race/Ethnicity	
Asian	18 (1)
Black	956 (40)
Indian	161 (7)
Mixed race	119 (5)
White	1062 (45)
Other	55 (2)
Annual household income^a^	
No income	115 (5)
Low income	379 (16)
Middle income	1030 (43)
High income	847 (36)
Employment status	
Student	1506 (63)
Unemployed and looking for work	66 (3)
Unemployed but not currently looking for work	10 (0.5)
Employed part‐time	144 (6)
Employed full‐time	547 (23)
Self‐employed	87 (4)
Receiving a pension	11 (0.5)
Receiving a grant	0 (0)
Unable to work	0 (0)
Marital status	
Single, never married	1861 (78)
Married or domestic partnership	448 (19)
Separated	13 (0.5)
Divorced	39 (2)
Widowed	10 (0.5)
Adult attachment style^b^	
Secure	1824 (77)
Dismissing (Insecure)	246 (10)
Preoccupied (Insecure)	172 (7)
Fearful (Insecure)	129 (6)

^a^ Annual household income categorized was classified according to Statistics South Africa (2011).

^b^ Adult attachment style was assessed using the Experience in Close Relationships—Relationship Structures (ECR‐RS) Questionnaire (Fraley et al., 2011).

Univariate models were run between chronic pain presence and various demographic and psychosocial variables (including adult attachment style). These univariate associations are summarized in Table 2. Presence of chronic pain was significantly associated with gender (being a woman), adult attachment styles (specifically, having an insecure attachment), household income (specifically, having a lower income), and increased depression, anxiety and pain catastrophizing. The reference variables for the univariate logistic regression models were “Men” (for gender), “Secure” (for the adult attachment style) and “No income” (for annual household income).

**TABLE 2:** Factors associating with the presence of chronic pain in the 2371 survey respondents.

	No chronic pain (*N* (%))	Chronic pain (*N* (%))	Odds ratio (95%CI)	*p*‐value
Total	1736 (73)	635 (27)	–	–
Gender				
Men	457 (77)	139 (23)	–	–
Women	1259 (72)	488 (28)	1.27 (1.03–1.59)*	.**028** *
Other	20 (71)	8 (29)	1.32 (.53–2.95)*	.524*
AAS	129			
Secure	1400 (77)	424 (23)	–	–
Dismissing	170 (69)	76 (31)	1.48 (1.10–1.97)**	.**009** **
Preoccupied	100 (58)	72 (42)	2.38 (1.72–3.27)**	**<.001** **
Fearful	66 (51)	63 (49)	3.15 (2.19–4.53)**	**<.001** **
AHI	847			
No income	74 (64)	41 (36)	–	–
Low income	241 (64)	138 (36)	1.03 (.67–1.61)***	.882***
Middle income	742 (72)	288 (28)	.70 (.47–1.06)***	.085***
High income	679 (80)	168 (20)	.45 (.30–.68)***	**<.001** ***

	No chronic pain (median [IQR])	Chronic pain (median [IQR])	Incidence rate ratio (95%CI)	*p*‐value
Age	23 [20–28]	23 [21–31]	1.06 (1.03–1.10)	.**001**
Depression	10 [4–20]	18 [8–28]	1.43 (1.34–1.52)	**<.001**
Anxiety	8 [4–16]	16 [8–24]	1.59 (1.49–1.69)	**<.001**
Pain catastrophizing	14 [6–24]	24 [13–36]	1.56 (1.47–1.65)	**<.001**

*Note*: Bolded = *p* < .05.

Abbreviations: AAS, Adult attachment style; AHI, Annual household income.

* Association when compared to men.

** Association when compared to the secure adult attachment style.

*** Association when compared to the no‐income group.

### Primary analysis: Is there an association between adult attachment style and chronic pain presence?

To determine the association between adult attachment style and chronic pain presence, two multiple logistic regression models were run. Table 3 shows the results of these regressions with secure as the reference attachment style. The first model investigates a total effect of adult attachment on chronic pain.

**TABLE 3:** Multivariable analyses showing the total effect of adult attachment style on chronic pain in our total sample (*N* = 2371).

AAS	Total effect^a^	
	Adjusted odds ratio (95%CI)	*p*‐value
Secure	–	–
Dismissing	1.38 (1.02–1.85)	.**037**
Preoccupied	2.26 (1.62–3.13)	**<.001**
Fearful	2.95 (2.03–4.29)	**<.001**

*Note*: Bolded = *p* < .05.

Abbreviations: AAS, Adult attachment style relative to secure attachment style.

^a^ Multivariable model including confounders and covariates, but excluding colliders and mediators.

### Secondary analysis: Is there an association between adult attachment style and the burden of chronic pain?

The secondary analysis explored the burden of pain (through the number of pain sites, pain severity and pain interference) for the four different attachment styles using the results of the BPI. Two hundred and eight individuals reported having chronic pain but did not complete the BPI‐sf. Thus, a total of 427 individuals (those with chronic pain who completed the BPI‐sf questionnaire) were included in the secondary analysis. A dropout analysis showed the following associations: individuals were more likely to drop out when they were male (compared to female) (odds ratio [CI], .64 [.43–.94], *p* = .024) with significantly lower depression (odds ratio [CI], .97 [.96–.99], *p* < .001), anxiety (odds ratio [CI], .96 [.94–.97], *p* < .001), and pain catastrophizing (odds ratio [CI], .99 [.98–1.00], *p* = .037).

**FIGURE 3:**
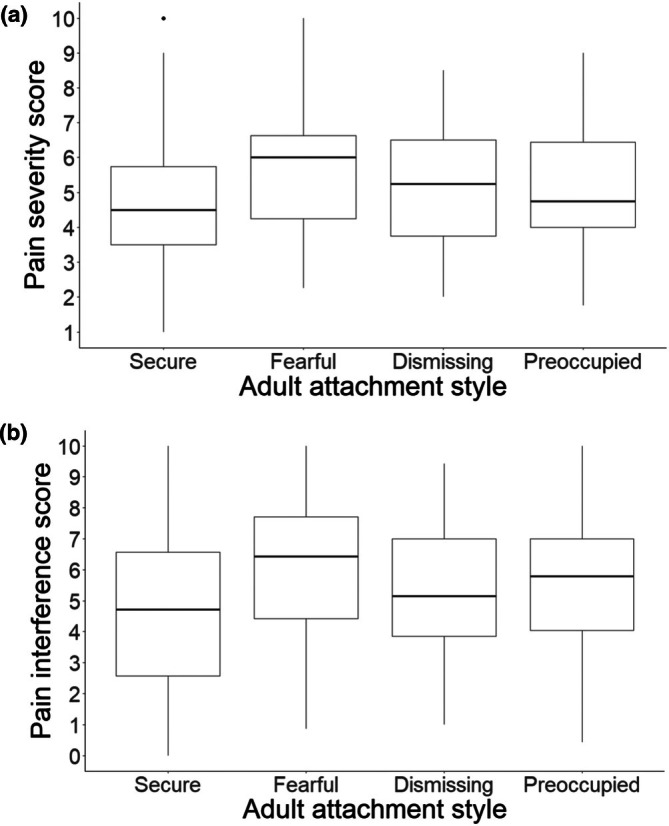
Pain severity score (Panel a) and Pain Interference Score (Panel b) in the last week for the four different adult attachment styles in individuals with chronic pain who completed the Brief Pain Inventory—short form (BPI‐sf) (*N* = 427).

The pain severity and pain interference scores for the adult attachment styles are summarized in Figure 3. Two separate univariate linear regression models were run for adult attachment with pain severity score and pain interference score. Compared to the secure attachment style, the dismissing (estimate [95%CI] = .48 [.00–.96], *p*‐value = .049) and fearful (estimate [95%CI] = .88 [.36–1.40], *p*‐value = .001) attachment styles significantly predicted increased pain severity scores. Pain interference scores were significantly higher in dismissing (estimate [95% CI] = .70 [.04–1.37], *p*‐value = .038), preoccupied (estimate [95% CI] = .93 [.23–1.63], *p*‐value = .010), and fearful (estimate [95%CI] = 1.31 [.59–2.03], *p*‐value < .001) attachment styles when compared to secure attachment. Multivariable analyses were run to determine the total effect of adult attachment style on pain severity and pain interference score. The fearful adult attachment style was associated with a total effect of increased pain severity score when compared to the secure attachment (estimate [95% CI] = .78 [.26–1.30], *p* = .004). There was also a total effect of all three insecure attachment styles being associated with worse pain interference scores when compared to the secure attachment style (dismissing: estimate [95% CI] = .73 [.06–1.41], *p* = .032; preoccupied: estimate [95% CI] = .88 [.17–1.58], *p* = .015; fearful: estimate [95%CI] = 1.17 [.44–1.90], *p* = .002).

With regard to the number of painful body sites in individuals reporting chronic pain, a univariate analysis showed that individuals with a preoccupied attachment had 1.32 times more pain sites (95% CI: 1.10–1.56, *p* = .002) and individuals with fearful attachment had 1.30 times more pain sites (95% CI: 1.09–1.55, *p* = .003) than individuals with secure attachment. Individuals with dismissing attachment did not have a significant difference in the number of pain sites compared to the securely attached individuals (IRR 1.02, 95% CI: .91–1.14, *p* = .733). After running multivariable analyses for the number of pain sites across the various attachment styles, there was a total effect whereby the fearful adult attachment style was associated with .78 times more pain sites compared to the secure attachment style (95% CI: .26–1.30, *p* = .003).

## DISCUSSION

Previous studies have assessed the association of attachment style with subsects of chronic pain, including chronic widespread pain and medically unexplained pain (Davies et al., 2009; McWilliams, 2017). This study is the first to assess the association between chronic pain presence and adult attachment style in a general population, with a standard definition of chronic pain. The order of most to least frequent attachment styles was the same as another student cohort from Ecuador using the fairly similar ECR‐R questionnaire (Díaz‐Mosquera et al., 2022). In our young, well‐educated and primarily female (74%) cohort, with a predominantly middle‐to‐high socioeconomic status, insecure attachment styles were associated with an increased presence of chronic pain. Both preoccupied and fearful styles were directly associated with the presence of chronic pain, and of note, the presence of chronic pain was more than double in individuals with a fearful attachment style compared to securely attached individuals. These data contribute to the emerging idea that a significant and clinically relevant relationship exists between attachment style and chronic pain.

Chronic pain prevalence is typically low in young people. In the United Kingdom, for example, the prevalence of chronic pain in a young (18–25 years) population is reported to be as low as 14% compared to the general prevalence that ranged from 35% to 51% over various studies in the United Kingdom with broader age ranges (Fayaz et al., 2016). A study conducted in the United States also found a lower prevalence of chronic pain (7.5%) in young adults (18–24 years) compared to the general population (21%). Previously published data in a national sample of South Africans, using the same question to determine chronic pain as we did, have demonstrated that 13% of 24–34 year olds have chronic pain (Kamerman et al., 2020), making the chronic pain prevalence of 27% in the present study surprisingly high. Our cohort was predominantly (74%) female, which may partially account for the higher‐than‐expected chronic pain prevalence, as women have higher localized and widespread chronic pain prevalences when compared to men (Racine et al., 2012; Umeda & Kim, 2019). In a study conducted on a South African cohort, the prevalence of chronic pain in women was 20% across all age groups (Kamerman et al., 2020). In the present study, the prevalence of chronic pain was 23% in the securely attached individuals, but 39% in individuals with insecure attachment styles. Indeed, specifically within the insecurely attached individuals, 49% of those with a fearful attachment had chronic pain. What is remarkable is that these high prevalence rates of chronic pain were evident in a cohort with an average age in the early twenties (median age 23 years; IQR 20–28) and predominantly from middle and high socioeconomic statuses (Prego‐Domínguez et al., 2021).

Our study here is the first to report that in a general population, attachment style is associated with chronic pain presence. The association between insecure attachment and chronic pain makes sense given the overlapping neurocircuitry both between physical and social pain (Eisenberger et al., 2003, 2006), and also between experimental pain perception and expectations of social safety or threat (Eisenberger et al., 2011). For example, when one perceives an environment and/or stimulus as threatening, the reported intensity of experimental pain is increased (Karos et al., 2020; Vlaeyen et al., 2009; Wang et al., 2016). Furthermore, there are associations between insecure attachment styles and factors associated with pain, including poorer mental health (Andersen, 2012; Ciechanowski et al., 2003; Kowal et al., 2015), greater number of sleep disruptions, and greater levels of inflammation (Adams & McWilliams, 2015; Gouin et al., 2009; Jaremka et al., 2013); all of which impact the perception of pain, whether the pain is experimental, acute, or chronic (Babiloni et al., 2020; Edwards et al., 2016). Indeed, early life experiences shape beliefs about safety and threat in a variety of situations, including response to pain, and data suggest that the perception of acute pain, the neurobiological and emotional responses to that pain, and the consequent behaviours (seeking of medical help or not) may predict whether an acute pain resolves or becomes chronic (Stamp et al., 2024).

Individuals with a fearful style had increased pain intensity, interference and number of pain sites compared to individuals with a secure attachment. However, no such relationship was found for dismissing and preoccupied styles. Only one study has investigated the association between the number of pain sites in chronic widespread pain and attachment style and found a greater number of pain sites in individuals with fearful, dismissing, and preoccupied styles (Davies et al., 2009). As an increased number of pain sites may be a sign of central sensitization (Hoshino et al., 2022; Inoue et al., 2025), an overactive state of the central nervous system that occurs with chronic pain (Latremoliere & Woolf, 2009), this association makes sense. As the Davies study (Davies et al., 2009) included a cohort of individuals with chronic widespread pain, they may have had greater central sensitization than our sample. It would be interesting for future studies to add a measure of central sensitization.

As the prevalence of chronic pain worldwide is 27.5% (ranging from 10% to 50%) (Zimmer et al., 2022), with a financial impact of $560 billion to $635 billion annually (Henschke et al., 2015), we believe that having a relatively simple way to predict who may be more likely to develop chronic pain is of interest to individuals working in public health and primary care. We also believe that important research questions include whether interventions for individuals with insecure attachment styles form part of trauma‐informed primary care and can prevent chronic pain from developing (Yamin et al., 2024). Future studies could, for example, extend preliminary data showing improvements in attachment style through psychological interventions, individually or in a group (Kinley & Reyno, 2013; Marmarosh & Tasca, 2013), or social interventions (e.g. supportive community groups, perhaps similarly designed to 12‐step recovery programmes) (Jordan, 2019) and determine whether proactive interventions could reduce the risk of developing chronic pain. Furthermore, the fearfully attached individuals, who were twice as likely to develop chronic pain here, may also be more likely to develop opioid addiction (Schindler et al., 2005, 2009) and so exploring interventions that could prevent the development of chronic pain could have multiple benefits for individuals, their family/friends, and also the larger community and economy.

### Study limitations

Certain limitations need to be acknowledged. Our data were collected from the second half of 2021, at the backend of the COVID‐19 pandemic. Given the evidence of increased levels of depression and anxiety, from German and Chinese populations, compared to pre‐pandemic levels (Bäuerle et al., 2020; Wu et al., 2021), the nature of our cohort may have been affected by the pandemic. We also recruited a convenience sample, that is, a sample of individuals responding to social media or email adverts to complete a survey on attachment style and pain. The cohort is therefore unlikely to be truly representative of the national population. This is further supported by the fact that respondents were 75% female, 99% had completed at least secondary education and all had access to a device and data with which to respond. This sex, education and socioeconomic profile is not representative of the South African population (Statistics South Africa, 2011), but it does make these data more generalizable to developed countries. The proportions of attachment styles may, therefore, not be representative of the general South African population. Another limitation was that 33% (208/635) of the individuals with chronic pain did not complete the BPI and so their pain intensity and interference data were missing. The dropout analysis indicated that there were 208 individuals with chronic pain who did not complete the BPI questionnaire and that these individuals were more likely to be male. The reasons for this are unclear. The single, standard question we used to determine chronic pain does not discriminate between primary pain, that is, pain with no identifiable cause, or secondary pain, where pain is a symptom of another condition or illness. Future work could extend this study and explore whether attachment style associates with primary and/or secondary pain. Other psychological factors not measured here may be associated with pain including neuroticism or cognitive inflexibility. Future studies could explore the mediating effect of such factors as potential targets for treatment.

Traditionally, the anxiety and avoidance subscales have been treated as orthogonal, thus opening the way to use the quadrant method of assigning attachment styles. The more recent method of analysing these two scales is to analyse them separately. That is, analyse the effect of anxiety on the odds of having chronic pain, and then perform the same analysis using avoidance as the predictor. Our scales were not orthogonal (Pearson's product–moment correlation = .56, *p* < .001), so it could be argued that we should have used the more recent approach to analysing these data. We feel, however, that performing separate analyses for the two scales misses out on what we as researchers wanted to know: the relationship between anxiety and avoidance, how they interact to determine attachment style, and how that style is related to the risk of having chronic pain.

## CONCLUSION

In this survey of over 2000 individuals, and the first to assess the association of attachment style and chronic pain in a general population with a standard definition of chronic pain, we found an association between insecure adult attachment styles and a greater presence of chronic pain. In addition, remarkably, the presence of chronic pain was more than double in individuals with an insecure fearful attachment style compared to those with a secure attachment style. Furthermore, the high presence of chronic pain in the insecure attachment styles (31–49%) was remarkable considering the cohort was young and had a high socioeconomic status. Individuals with all three insecure attachment styles had worse pain interference, and individuals with a fearful attachment style also had increased pain severity and more pain sites than securely attached individuals. Having found these associations in a cross‐sectional study, future longitudinal studies should map attachment security with the incidence and outcomes of chronic pain, to determine the possible vulnerability that individuals with an insecure adult attachment style have in developing chronic pain. These findings underscore a critical need to integrate attachment‐based assessments and interventions into public health strategies, with the potential to address chronic pain at its psychological and social roots.

## AUTHOR CONTRIBUTIONS

**Gabriella Elisabeth Stamp:** Conceptualization; investigation; writing – original draft; methodology; formal analysis; project administration; data curation; visualization; validation; software. **Stella Iacovides:** Conceptualization; funding acquisition; writing – review and editing; supervision; resources; methodology. **Antonia Louise Wadley:** Conceptualization; funding acquisition; writing – review and editing; supervision; resources; methodology.

## Data Availability

All data, analysis scripts and analysis script outputs are available at Figshare: https://doi.org/10.6084/m9.figshare.23531076.v1.
